# Ligand binding at the A-cluster in full-length or truncated acetyl-CoA synthase studied by X-ray absorption spectroscopy

**DOI:** 10.1371/journal.pone.0171039

**Published:** 2017-02-08

**Authors:** Peer Schrapers, Julia Ilina, Christina M. Gregg, Stefan Mebs, Jae-Hun Jeoung, Holger Dau, Holger Dobbek, Michael Haumann

**Affiliations:** 1 Department of Physics, Freie Universität Berlin, Berlin, Germany; 2 Institute of Biology, Structural Biology/Biochemistry, Humboldt-Universität zu Berlin, Berlin, Germany; Russian Academy of Medical Sciences, RUSSIAN FEDERATION

## Abstract

Bacteria integrate CO_2_ reduction and acetyl coenzyme-A (CoA) synthesis in the Wood-Ljungdal pathway. The acetyl-CoA synthase (ACS) active site is a [4Fe4S]-[NiNi] complex (A-cluster). The dinickel site structure (with proximal, p, and distal, d, ions) was studied by X-ray absorption spectroscopy in ACS variants comprising all three protein domains or only the C-terminal domain with the A-cluster. Both variants showed two square-planar Ni(II) sites and an OH^-^ bound at Ni(II)_p_ in oxidized enzyme and a H_2_O at Ni(I)_p_ in reduced enzyme; a Ni(I)_p_-CO species was induced by CO incubation and a Ni(II)-CH_3_^-^ species with an additional water ligand by a methyl group donor. These findings render a direct effect of the N-terminal and middle domains on the A-cluster structure unlikely.

## Introduction

Carbon oxide (CO_x_) conversion is a challenging task in renewable energy exploration, combating the atmospheric greenhouse effect, and chemical research aiming at new catalysts using abundant metal species [1, 2]. In nature, various enzymes are found, which catalyze efficient and reversible CO_x_ transformations at active sites binding nickel, iron, or molybdenum ions [3–7]. Several biological carbon dioxide (CO_2_) to biomass conversion pathways exist in prokaryotes and eukaryotes [8]. Various prokaryotes employ the Wood-Ljungdahl pathway to reductively form acetyl coenzyme-A [9, 10]. Carbon monoxide (CO) as obtained from CO_2_ reduction by CO dehydrogenase (CODH) is utilized in a reaction involving two enzymes, corrinoid iron-sulfur protein (CoFeSP) with a methyl group bound to the cobalt ion of its cobalamin cofactor and acetyl-CoA synthase (ACS), to produce acetyl-CoA, the central metabolic building block (Eq 1) [11–14].

$$ACS+CoFeSP\text{-}CH_{3}+CO+CoA\to ACS+CoFeSP+acetyl\text{-}CoA$$ (1)

**Fig 1:**
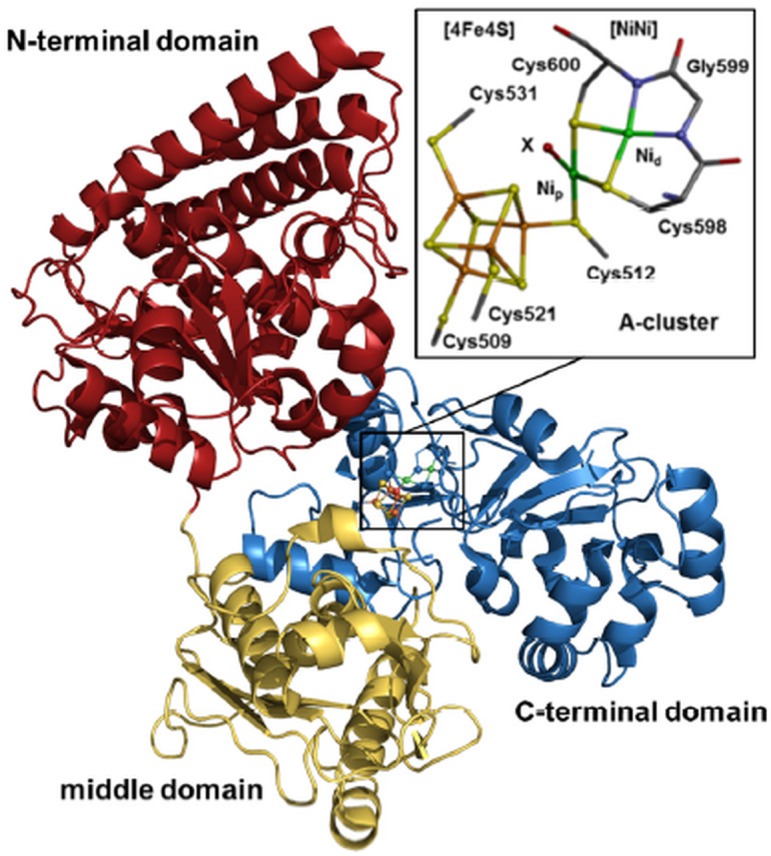
Crystal structure of ACS from *C*. *hydrogenoformans*. PDB entry 1RU3, 2.2 Å resolution [15]. Inset, A-cluster in magnification (color code: green, Ni; orange, Fe; yellow, S; red, O; blue, N; grey, C; protons are omitted for clarity). The (distorted) square-planar Ni ions are denoted proximal (p) and distal (d). X marks a ligand modelled as oxygen.

ACS is often found in association with the other enzymes involved in reaction (1): in bacteria, ACS forms a complex with CODH whereas in methanogenic archaea, ACS is part of an oligomeric complex comprising CODH and CoFeSP molecules. In contrast, when *Carboxydothermus hydrogenoformans* grows under carboxydothrophic conditions, its ACS (ACS_Ch_) is present as a monomeric enzyme [15]. Crystal structures of ACS alone and of its complex with CODH have revealed that the enzyme consists of three (N-terminal, middle, and C-terminal) domains connected by flexible linkers [15–17], with the C-terminal domain binding a unique iron-nickel complex denoted A-cluster (Fig 1). This cofactor consists of a canonical [4Fe4S] cluster, which is linked by a cysteine thiolate to the proximal nickel ion (Ni_p_) of the dinickel sub-complex. The [NiNi] center features unusual binding of the distal nickel ion (Ni_d_) to the backbone amide groups of a glycine and a cysteine residue and Ni-Ni bridging by two cysteine thiolates [15, 17]. A fourth ligand in equatorial position at Ni_p_ was assigned as an oxygen species or an unknown exogenous ligand [15, 17]. Cu and Zn can bind instead of nickel at the Ni_p_ site, thereby inactivating ACS [16, 18, 19]. Crystal structures of ACS with nickel-bound carbon monoxide or methyl groups are not available.

Previous experimental and theoretical investigations on ACS have suggested that Ni_p_ directly participates in acetyl-CoA formation, involving a divalent Ni_d_ ion and reversible Ni(I) formation (in the so-called “paramagnetic mechanism”) [2, 7] or Ni(0) formation (“diamagnetic mechanism”) from a divalent Ni_p_ [20]. According to the paramagnetic mechanism, CO accesses the A-cluster via a channel [16] and can bind to Ni_p_ to form a Ni(I)-CO species, which upon reduction accepts an additional methyl group, so that subsequent C-C bond formation and S-C coupling with a CoA molecule finally yields acetyl-CoA [6, 14, 21–24]. The influence of the N-terminal and middle ACS domains on the function of the A-cluster has remained a matter of debate [14, 17, 25, 26]. Interestingly, acetyl-CoA synthesis activity of ACS under in vitro conditions depends on the relative CO concentration. Full-length ACS_Ch_ shows maximal activity with a methyl group donor at sub-stoichiometric CO concentrations and loses activity at (super-)stoichiometric CO concentrations [26]. This apparent substrate inhibition may be due to binding of a second CO molecule at nickel, generating an inactive species, and/or to the sequence of CO and methyl group binding steps at the A-cluster. In the absence of the N-terminal domain, diminished activity at low CO concentrations, but, in contrast to full-length ACS_Ch_, highest activity at saturating CO concentrations was reported [26]. These results may suggest that the N-terminal domain directly affects the structure of the active site A-cluster, possibly by influencing the location or orientation of bound substrate.

X-ray absorption spectroscopy (XAS) facilitates monitoring of redox and geometry changes, as well as determination of precise interatomic distances at protein-bound metal centers [27, 28]. Relatively few earlier XAS studies at iron and nickel K-edges on ACS proteins have revealed structural parameters in agreement with more recent crystallographic data for oxidized ACS [15, 18, 29, 30]. CO binding and nickel and iron reduction have been detected as well and square-planar or tetrahedral nickel sites and typical [4Fe4S] motifs were suggested [30]. The presence of high- and low-spin Ni(II) sites in oxidized and Ni(I) formation in reduced enzyme was concluded from a Ni L-edge study [31]. These results have significantly contributed to our understanding of the structure and function of the A-cluster, but did not fully clarify relations between nickel redox states and site geometries. The influence of the N-terminal domain on the A-cluster structure has not been studied by XAS.

Here, XAS at the Ni K-edge was used to characterize ACS variants from *C*. *hydrogenoformans* containing the complete protein (three domains, ACS_NMC_) or only the C-terminal domain (ACS_C_), which were poised in oxidized or reduced states under conditions facilitating CO or methyl group binding to the active site. Our analysis shows that the N-terminal and middle domains do not affect the A-cluster structure or nickel reduction and ligand binding, suggests two square-planar Ni(II) sites in oxidized ACS and a Ni(I) ion in reduced ACS, and favors replacement of an equatorial OH^-^ by a CO group, but binding of the methyl ligand in addition to the water ligand.

## Materials and methods

### Protein sample preparation

Complete ACS protein from *Carboxydothermus hydrogenoformans* (Fig 1) comprising the N-, middle, and C-terminal domains (ACS_NMC_) and a truncated protein variant comprising only the C-terminal domain (ACS_C_) binding the A-cluster were heterologously expressed in *Escherichia coli* BL21 (DE3) in mTB media via a pET28-twinStrep-TEV-vector adopting previously established protocols [32]. The pET28-twinStrep-TEV-vector was constructed by introducing a twin strep tag with a tobacco etch virus (TEV) protease cleavage site and replacing the His-tag of the commercially available pET28a vector (Novagen) [33]. Cells were grown under aerobic conditions to an optical density at 600 nm (OD_600_) of 0.6 ± 0.1 at 37°C, and were then induced with 0.5 mM isopropyl-β-D-thiogalactopyranosid (IPTG) and transitioned to anoxic conditions. Cells were harvested approximately 20 h after induction. Protein purification, reconstitution, and XAS sample preparation were carried out under anoxic conditions in a 95% N_2_ and 5% H_2_ atmosphere at room temperature in a glove box. The protein was purified by subsequent use of Strep-Tactin and S200 columns (lysis/ running/ washing buffer: 50 mM Tris-HCl pH 8.0, 100 mM NaCl, 2 mM tris(2-carboxyethyl)phosphin (TCEP); elution buffers: Strep-Tactin column: 50 mM Tris-HCl pH 8.0, 100 mM NaCl, 2 mM TCEP, 2.5 mM desthiobiotin; S200 column: 50 mM Tris-HCl pH 8.0, 100 mM NaCl, 2 mM TCEP). For nickel reconstitution, 250 μM NiCl_2_ was added to a solution of 100 μM purified ACS protein and the mixture was incubated for 48 h (samples denoted ox_NMC/C_). ACS was reduced (red_NMC_) by addition of 300 μM Ti(III)-citrate to a protein solution (100 μM) and incubation for 20 min. Protein with cofactor modifications was produced from reduced ACS (100 μM) by incubation in a carbon monoxide saturated (~1 mM CO) buffer (red_NMC_^CO^_/C_^CO^) or in a buffer containing 0.9 mM methyl-cobinamide (red_NMC_^Me^_/C_^Me^). Proteins were concentrated to ~1 mM (Vivaspin 500, 10 kDa cut-off) as determined using the Bradford method, 10% (v/v) glycerol was added as a cryo-protectant, and samples (30 μl) were filled into Kapton-covered acrylic-glass holders for XAS and immediately frozen in liquid nitrogen.

### Metal content determination

Element quantification in ACS samples was carried out by total-reflection X-ray fluorescence analysis (TXRF) [34] on a PicoFox instrument (Bruker) after addition of a Ga elemental standard (Sigma-Aldrich, 1/1 v/v). TXRF spectra were analyzed using the routines provided with the spectrometer.

### X-ray absorption spectroscopy

XAS at the Ni K-edge was performed at BESSY (Helmholtz-Center for Materials and Energy, Berlin, Germany) at beamline KMC-1. Kα-fluorescence detected XAS spectra were collected using an energy-resolving 13-element Ge detector (Canberra) shielded by 10 μm Co foil against scattered X-rays and a Si[111] double-crystal monochromator (energy calibration by the K-edge inflection at 8333 eV of a Ni foil measured simultaneously in transmission mode) in a standard XAS set-up on samples held at 20 K in a liquid-He cryostat (Oxford) [11, 35]. Up to 10 detector deadtime-corrected XAS scans were averaged for signal-to-noise ratio improvement. XAS data processing for XANES normalization and EXAFS extraction was carried out as previously described [27]. *k*^3^-weighted EXAFS spectra were simulated using the in-house software SimX [27] and phase functions calculated with FEFF9 (S_0_^2^ = 0.9) [36]. Fourier-transforms (FTs) of EXAFS spectra were calculated for *k* = 1.8–13.1 Å^-1^ (cos windows extending over 10% of both *k*-range ends).

## Results

### Metal content

TXRF analysis of metal contents in Ni-reconstituted ACS samples yielded the concentrations listed in Table 1 (see Materials and Methods for preparation and annotation of oxidized (ox) or reduced (red) full-length (NMC) or truncated (C) enzyme samples). On average ~4.5 Fe ions per full-length ACS protein in ox_NMC_ and red_NMC_ samples within error limits suggested near-stoichiometric loading with the [4Fe4S] sub-complex of the A-cluster. Apparently higher mean iron contents (~5.9 Fe ions per ACS C-terminal domain) in ox_C_ and red_C_ samples presumably reflected moderate underestimation of the protein concentration so that four Fe ions likely were also present in the truncated variant. The mean Ni/Fe ratio of ~0.4 (~0.3) in ox/red_C_ (ox/red_NMC_) samples, which was slightly below the ideal value of 0.5 (for 2 Ni in the dinickel site and 4 Fe in the [4Fe4S] sub-complex), and the lower Zn/Fe ratio (~0.2) implied that maximally ~80% (~60%) of ACS proteins contained a [NiNi] site and the remainder an unoccupied binding pocket, only one Ni ion, or a [NiZn] site [30]. This may suggest somewhat more efficient nickel reconstitution in the truncated vs. the full-length ACS variant.

**Table 1:** Metal content of ACS_Ch_ samples from TXRF.^a^

ACS_Ch_	concentration [mM] (metal per protein)			ratio	
sample	Fe	Ni	Zn	Ni/Fe	Zn/Fe
ox_NMC_	5.2 (4)	1.5 (1.2)	1.1 (0.8)	0.3	0.2
ox_C_	6.5 (4)	2.7 (1.7)	1.2 (0.7)	0.4	0.2
red_NMC_^Me^	4.3 (4)	1.5 (1.4)	0.9 (0.8)	0.3	0.2
red_C_^Me^	5.9 (4)	2.3 (1.6)	0.9 (0.6)	0.4	0.2
red_NMC_^CO^	4.4 (4)	1.4 (1.3)	1.0 (0.9)	0.3	0.2
red_C_^CO^	5.2 (4)	1.9 (1.5)	0.8 (0.6)	0.4	0.2
red_NMC_	4.3 (4)	1.5 (1.4)	1.0 (0.9)	0.3	0.2

^a^Concentrations (error ±0.5 mM) are for oxidized (ox) or reduced (red) Ni-reconstituted ACS_Ch_ variants comprising the N-, middle, and C-terminal domains (NMC) or only the C-terminal domain (C) and the indicated cofactor modifications (CO = carbon monoxide treatment, Me = methyl-cobinamide treatment). Values in parenthesis show metal-to-protein ratios calculated under the assumption that four iron ions in the [4Fe4S] cluster are present in both ACS_Ch_ variants irrespective of the occupation of the nickel binding sites. The protein concentration was estimated as 1.0±0.2 mM in all samples.

### XANES of ACS

**Fig 2:**
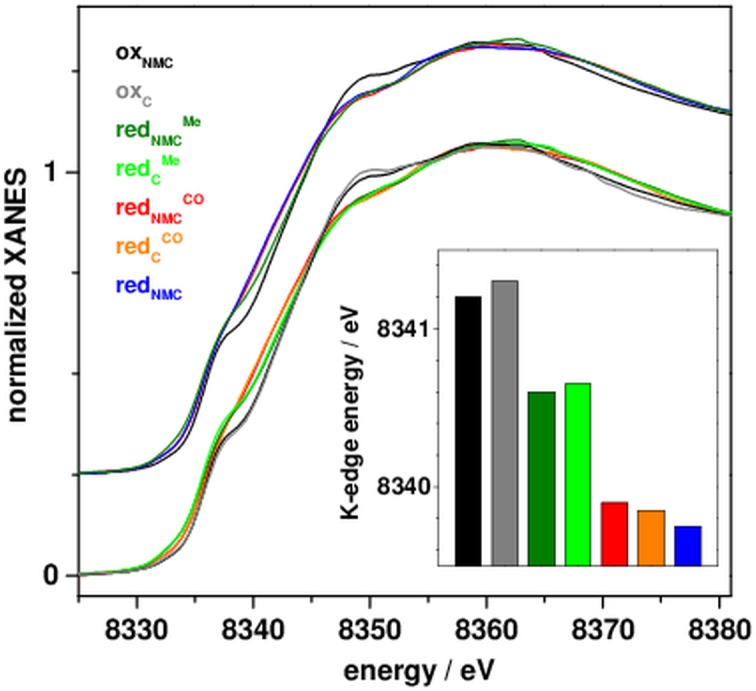
Ni XANES spectra of ACS_Ch_. Spectra of the indicated protein samples in the main panel were in part vertically displaced for comparison (dashes mark zero levels), the inset shows respective K-edge energies (at 50% level of normalized X-ray absorption).

X-ray absorption spectra of ACS samples at the Ni K-edge are shown in Fig 2. Similar edge spectra of oxidized and reduced samples and comparable shape changes in the CO/Me proteins suggested a largely analogous nickel coordination and similar effects of the treatments in the full-length and truncated variants. The low primary edge maximum and shallow edge slope of all spectra indicated preferential sulfur binding to nickel in low coordination environments [30, 35, 37, 38] as found in the crystal structures [15–17]. The mean edge energy (~8341.2 eV) and the edge shoulder (~8338 eV) of the oxidized proteins suggested the predominant presence of square-planar (low-spin) Ni(II) species [30, 39]. Reduced ACS showed a ~1.5 eV lower mean edge energy (~8339.7 eV), suggestive of formation of one Ni(I) ion [30, 38, 40]. The less pronounced shoulder in the K-edge may suggest a geometry change, possibly towards a more tetrahedral ligand arrangement at the Ni(I) ion [15]. Significant edge shape changes were observed for the reduced CO- and Me-treated proteins. For red_NMC_^CO^_/C_^CO^, the edge energy and shape were similar to red_NMC/C_, meaning that potential carbonyl binding to nickel (i.e. replacing the equatorial ligand X at Ni_p_) may not affect the XANES significantly. For red_NMC_^Me^_/C_^Me^, a ~0.9 eV higher edge energy compared to red_NMC_^CO^_/C_^CO^, but less pronounced edge shoulder compared to red_NMC/C_ was observed, indicated a geometry change at nickel, possibly due to binding of a methyl group (e.g. as a fifth ligand at Ni_p_).

### EXAFS analysis

**Fig 3:**
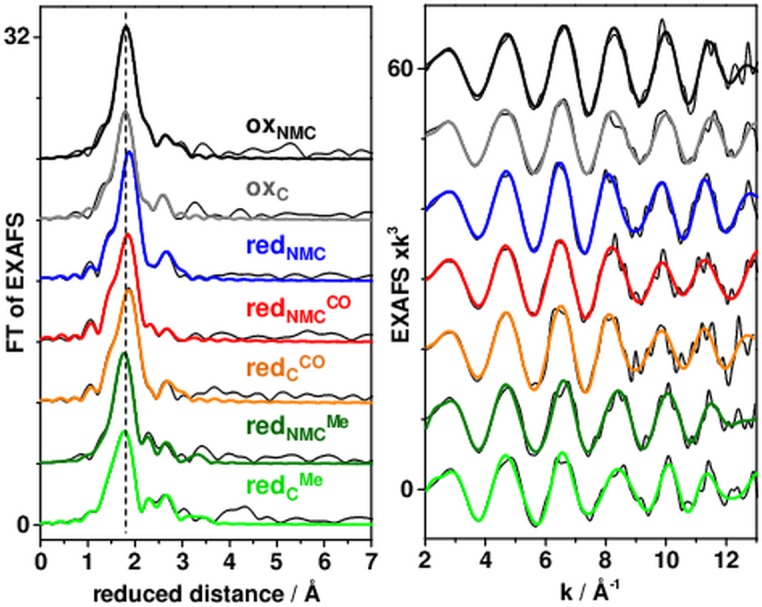
EXAFS analysis of ACS_Ch_. Thin black lines, experimental data (vertically shifted for comparison); thick (colored) lines, simulation curves with parameters in Table 2 (second fits). Vertical dashes highlight the Fourier-transform (FT) main peak position.

EXAFS spectra of the ACS samples are shown in Fig 3. All Fourier-transform (FT) spectra revealed overall similar shapes featuring a main FT peak at ~1.8 Å of reduced distance reflecting the Ni-N/O/S bonds. Small FT features at higher distances likely were due to interfering contributions from Ni-Ni and Ni-/Fe distances. Similar spectral features were observed for the full-length and truncated oxidized ACS variants, in agreement with the XANES data. For red_NMC/C_ samples, the main FT peak was shifted to slightly larger distances compared to ox_NMC/C_, which suggested bond elongation at more reduced nickel sites. For red_NMC_^CO^_/C_^CO^, a similarly up-shifted peak and a more pronounced low-distance shoulder likely were due to a shorter Ni-C(O) bond. For red_NMC_^Me^_/C_^Me^, the smaller and somewhat broader main FT peak may suggest overlaid contributions of Ni-N/S and (longer) Ni-C(H_3_)/O bonds.

**Fig 4:**
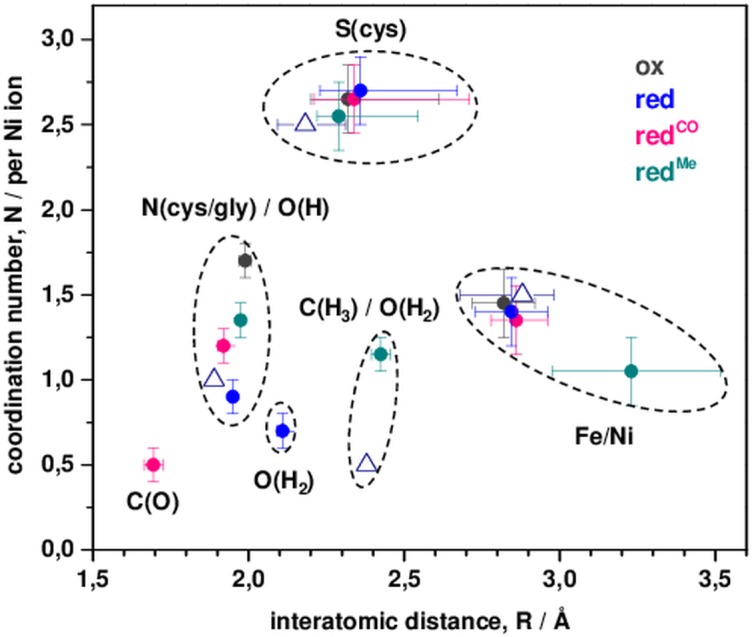
Nickel coordination changes from EXAFS. Mean *R*-values (weighted by the coordination numbers) and (summed) *N*-values for NMC and C variants (Table 2) are shown for ox, ox^CO^, and ox^Me^ samples. x-error bars show full distance ranges for Ni-S and Ni-Ni/Fe shells or estimated EXAFS fit errors for the other shells; y-error bars show the estimated maximal fit error. Assignment of parameters to C(O), O(H), O(H_2_), and C(H_3_) ligands is tentative but plausible; see Fig 1 for N/S ligands from amino acid groups. Open triangles, data for crystal structure 1RU3 [15] of oxidized ACS_Ch_ (the long Ni_p_-O distance of ~2.74 Å was omitted; the triangle at a distance of ~2.4 Å corresponds to the Ni-ligand bond in structure 1OAO [17]).

EXAFS simulations revealed nickel-ligand bond lengths and metal-metal distances in the ACS samples (Table 2). Already the coordination numbers (*N*) from the crystallographic data of the A-cluster (Fig 1) described the EXAFS spectra of ox_NMC/C_ quite well (R_f_ < 10%). The analysis revealed Ni-O/N (~2.0 Å), Ni-S (~2.2 Å), Ni-Ni (~2.9 Å), and Ni-Fe (~2.7 Å) distances (*R*), in good agreement with two square-planar nickel sites and a relative orientation of the [NiNi] and [4Fe4S] sub-complexes as in the crystal structure, showing a Ni-Ni distance larger than the Ni-Fe distance to the closest iron ion of the [4Fe4S] cluster (Fig 4). However, a fit approach with variable *N*-values showed slightly larger numbers of Ni-S bonds and a smaller number of Ni-Ni distances and a larger number of Ni-Fe distances compared to the ideal coordination numbers. This result is in agreement with the sub-stoichiometric metal contents from TXRF and suggested a minor fraction of centers with an unoccupied metal binding site in the samples. Destructive interference of EXAFS oscillations from Ni-Ni, Ni-Fe, and similarly long Ni-S and Ni-C distances (i.e. of the S-atom in the [4Fe4S] cluster closest to Ni_p_ and of second-sphere Ni-C distances of amino acids) explained the relatively small amplitude contributions from metal-metal distances to the FT spectra [41, 42].

**Table 2:** EXAFS simulation parameters.^a^

ACS_Ch_	fit	N [per Ni ion] / R [Å] / 2σ^2^ x10^3^ [Å^2^]			R_f_
sample	no.	Ni-C/N/O	Ni-S	Ni-Ni/Fe/Zn	[%]
ox_NMC_	1	1.5* / 2.00 / 7	1.9^#^ / 2.20 / 5	1.0* / 2.91 / 11	8.0
			0.6^#^ / 2.60 / 4	0.5* / 2.70 / 1	
	2	1.8 / 2.00 / 9	1.8 / 2.20 / 5*	0.8 / 2.91 / 5*	5.9
			0.9 / 2.61 / 5*	0.7 / 2.70 / 5*	
ox_C_	3	1.5* / 1.99 / 5	1.8^#^ / 2.22 / 5	1.0* / 2.93 / 15	7.7
			0.7^#^ / 2.62 / 3	0.5* / 2.69 / 3	
	4	1.6 / 1.98 / 6	1.9 / 2.22 / 5*	0.7 / 2.93 / 5*	7.2
			0.7 / 2.63 / 5*	0.7 / 2.72 / 5*	
red_NMC_	5	1.5* / 1.99 / 10	2.0^#^ / 2.24 / 5	1.0* / 2.97 / 21	9.1
			0.5^#^ / 2.58 / 1	0.5* / 2.63 / 8	
	6	0.9 / 1.95 / 2^&^	1.9 / 2.23 / 5*	0.7 / 2.96 / 5*	6.6
		0.7 / 2.11 / 2^&^	0.8 / 2.67 / 5*	0.7 / 2.73 / 5*	
red_NMC_^CO^	7	1.5* / 1.98 / 3	1.4^#^ / 2.24 / 2	1.0* / 2.81 / 36	16.4
			1.1^#^ / 2.51 / 24	0.5* / 2.60 / 16	
	8	1.1 / 1.90 / 2	2.0 / 2.20 / 5*	0.7 / 2.97 / 5*	4.6
		0.6^&^ / 1.68 / 2*	0.6 / 2.75 / 5*	0.8 / 2.81 / 5*	
		0.6^&^ / 3.11 / 2*			
red_C_^CO^	9	1.5* / 1.98 / 5	1.6^#^ / 2.25 / 3	1.0* / 2.96 / 28	12.2
			0.9^#^ / 2.58 / 21	0.5* / 2.67 / 22	
	10	1.3 / 1.94 / 3	1.9 / 2.22 / 5*	0.6 / 2.94 / 5	6.6
		0.4^&^ / 1.71 / 2*	0.8 / 2.67 / 5*	0.6 / 2.74 / 5*	
		0.4^&^ / 3.12 / 2*			
red_NMC_^Me^	11	1.5* / 2.02 / 6	2.1^#^ / 2.19 / 13	1.0* / 2.92 / 18	11.1
			0.4^#^ / 2.49 / 46	0.5* / 2.70 / 5	
	12	1.4 / 1.99 / 3	1.7 / 2.22 / 5*	0.6 / 2.97 / 5*	6.2
		1.1 / 2.46 / 2*	0.9 / 2.45 / 5*	0.5 / 3.49 / 5*	
red_C_^Me^	13	1.5* / 1.97 / 8	2.0^#^ / 2.20 / 11	1.0* / 2.95 / 16	10.6
			0.5^#^ / 2.51 / 29	0.5* / 2.67 / 8	
	14	1.3 / 1.96 / 5	1.8 / 2.22 / 5*	0.5 / 2.98 / 5*	6.5
		1.2 / 2.39 / 2*	0.7 / 2.42 / 5*	0.5 / 3.54 / 5*	

^a^Data correspond to spectra in Fig 3. *N* = coordination number, *R* = interatomic distance, 2σ^2^ = Debye-Waller parameter, R_f_ = fit error sum (calculated for reduced distances of 1–3 Å).

*Parameters that were fixed (to chemically reasonable values) in the fit procedure

^#^sulfur coordination numbers were coupled to yield a sum of 2.5 accounting for the mean value over both nickel sites in the [NiNi] sub-complex (Fig 1)

^&^coordination numbers were coupled to yield the same values for the two shells.

**Fig 5:**
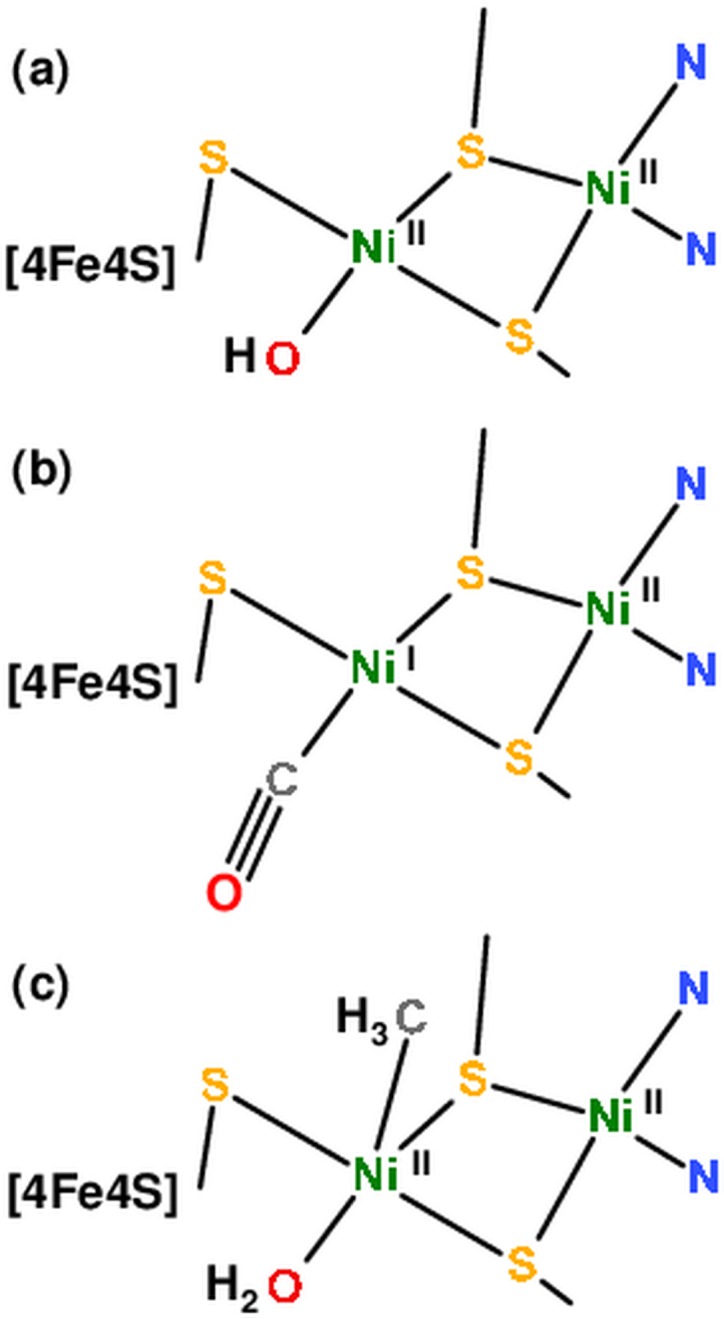
Structural models for [NiNi] sites in ACS_Ch_. Overall geometries correspond to crystal structure 1RU3 [15], ligand assignments agree with our XAS analysis. (a) Oxidized A-cluster with two square-planar Ni(II) ions. Ni(I)_p_ presumably is formed in reduced enzyme, leading to protonation of the equatorial OH^-^ to a (neutral) water ligand. (b and c) Reduced enzyme with Ni_p_-bound (equatorial) carbon monoxide or with (apical) methyl and (equatorial) water ligands. Tentative assignment of the proximal site as Ni(II) in the methyl/water-bound state of reduced enzyme may imply reduction of the [4Fe4S] cluster by charge transfer from the [NiNi] site [30]. We note that oxidation state assignments are formal. Further nickel site geometry distortion (i.e. towards more tetrahedral ligand arrangements) in reduced proteins may not be excluded, but is not uniquely implied by our XAS data.

Reduced ACS (red_NMC/C_) showed ~0.05 Å longer main Ni-S distances in addition to a diminished coordination number of short Ni-N/O bonds and a superior fit quality was obtained by inclusion of a longer Ni-O bond (~2.1 Å). The Ni-Ni/Fe distances were almost unchanged compared to oxidized ACS. These results suggested two nickel sites of overall similar geometry also in reduced ACS, one of which likely was reduced to the Ni(I) level, but a bound equatorial water (H_2_O) ligand at Ni_p_ (with a longer bond) instead of a hydroxyl group (OH^-^) in the oxidized protein. For both red_NMC_^CO^_/C_^CO^ and red_NMC_^Me^_/C_^Me^, the EXAFS fit approach with crystallographic coordination numbers yielded unsatisfactory results (R_f_ > 10%), but inclusion of additional ligands provided an about two-fold increased fit quality (Table 2). For red_NMC_^CO^_/C_^CO^, a diminished *N*-value of Ni-O bonds and a shorter nickel-ligand bond (~1.7 Å) were found (Fig 4). We attribute the short distance to a carbonyl ligand, which presumably replaces the equatorial water species at Ni_p_ in red_NMC_^CO^_/C_^CO^, thereby resulting in a similar overall nickel site structure as in the reduced sample. The even smaller FT peaks due to Ni-Ni/Fe distances were explained by further interference with (multiple scattering) EXAFS contributions from the oxygen atom of the CO ligand at ~3 Å to Ni_p_ [43]. For red_NMC_^Me^_/C_^Me^, diminished *N*-values of shorter Ni-N/O bonds, about two longer Ni-N/O bonds (~2.4 Å), and significantly larger Ni-Ni/Fe distances provided superior fit qualities (Table 2). These findings suggest that the equatorial oxygen ligand was a neutral water rather than a hydroxyl species as in ox_NMC/C_ and that a methyl group (CH_3_) may bind as a fifth (apical) ligand at Ni_p_, which leads to a larger distance in particular of Ni_p_ to the closest iron ion of the cubane cluster. Such an arrangement may further result in a (formal) Ni(II) species in red_NMC_^Me^_/C_^Me^. We summarize our structural attributions for A-cluster species from XAS schematically in Fig 5.

## Discussion

XAS analysis of *C*. *hydrogenoformans* ACS has revealed significant structural changes at the [NiNi] sub-complex of the A-cluster upon enzyme reduction and treatment with carbon monoxide or a methyl group donor. However, similar structural features were detected in ACS_Ch_ variants comprising the N-, middle, and C-terminal domains or only the C-terminal domain. The three domains are connected by a flexible linker, which facilitates large-scale conformational changes leading to “open” or “closed” configurations of the enzyme in CODH/CoFeSP/ACS protein complexes [14–17, 44, 45]. Our results suggest that the A-cluster structure and the ability for cofactor reduction, as well as for carbon monoxide and methyl ligand binding are unrelated to the presence of the N-terminal and middle domains in ACS_Ch_. Significant structural variations at the A-cluster due to relative orientation changes of the protein domains in the protein complexes thus may not be expected. However, we generated the reduced and CO-binding enzyme species under saturating CO concentrations, at which full-length ACS_Ch_ is inhibited by CO, but highest activity was reported for an ACS_Ch_ variant without N-terminal domain [25]. We found that the coordination geometry at nickel, including binding of a single CO ligand, was similar in the presence or absence of the N-terminal domain of ACS_Ch_. Rather than binding of two CO molecules, conformational changes due to domain movements not affecting the bound CO, but possibly the relative rate and/or sequence of CO and methyl binding events, may explain the lower catalytic activity at high CO concentrations in full-length compared to truncated ACS_Ch_ [26].

The XAS parameters for the oxidized ACS_Ch_ support a [NiNi] site geometry with two square-planar nickel ions as visible in crystal structures [15, 17] and suggest a Ni(II) oxidation state for both. This assignment agrees with previous spectroscopic studies and theoretical studies (see, e.g., refs. [15, 18, 29–31, 46–47]). Our data further support an arrangement of the [4Fe4S] and [NiNi] complexes with a Ni-Ni distance shorter than the closest Ni-Fe distance. Ti(III)-citrate treatment of ACS_Ch_ causes significant nickel reduction, which we interpret as formation of a single Ni(I) ion, in line with previous findings [23, 30]. Detection of elongated Ni-S bonds and replacement of a short by a longer Ni-O bond can be explained by protonation of a hydroxyl group at nickel (Ni(II)-OH^-^) in oxidized ACS_Ch_ to become a water ligand (Ni(I)-OH_2_) in reduced enzyme. An oxygen species was resolved in equatorial position at Ni_p_ by crystallography [15], which we hence attribute to the respective water species, in turn suggesting that the proximal nickel is reduced. Otherwise, the overall cofactor configuration in oxidized and reduced ACS_Ch_ is quite similar, which does not exclude distortion (i.e. towards tetrahedral symmetry) in particular of the Ni_p_ site in reduced enzyme [30, 31].

Significant structural changes at one of the nickel ions were detected both for CO- and methyl-treated ACS_Ch_. The relative arrangement of the [4Fe4S] and [NiNi] sub-complexes in the crystal structures precludes axial ligand binding at Ni_p_ and Ni_d_ below the equatorial plane (Fig 1). Axial binding of the ligand above the equatorial plane of Ni_d_ and Ni_p_ would require movement of a phenylalanine residue (Phe515 in ACS_Ch_) as observed in CODH/ACS structures [15, 17]. The XAS parameters for CO-treated ACS suggested that the carbonyl replaces the equatorial water species rather than binding as a surplus (apical) ligand to Ni_p_. Apparently, such CO binding otherwise causes only minor structural changes at the cofactor. These results support assignment of the formed Ni(I)-CO species to the Ni_p_ site [48].

A different situation was found for methyl-treated ACS, for which binding of a methyl group in addition to the water species is suggested. Strictly speaking, the XAS data is not decisive whether the methyl (fifth) ligand is bound at Ni_p_ or Ni_d_, but suggests that the site of binding is described by a (formal) Ni(II)-CH_3_^-^ species, possibly due to charge transfer from the methylated nickel to the [4Fe4S] cluster [30, 48, 49]. For binding of a methyl and a water ligand at the same nickel ion, a catalytic intermediate with simultaneous CO and methyl binding at Ni_p_, as postulated in both mechanistic proposals [2, 7, 20], seems to gain probability.

## Abbreviations

ACS: acetyl-CoA synthase
EXAFS: extended X-ray absorption fine structure
TXRF: total-reflection X-ray fluorescence analysis
XANES: X-ray absorption near edge structure
XAS: X-ray absorption spectroscopy
